# Supplementary material for: Inflammatory factors and risk of lung adenocarcinoma: a Mendelian randomization study mediated by blood metabolites
Source: Front Endocrinol (Lausanne). 2024 Aug 27;15:1446863. doi: 10.3389/fendo.2024.1446863 (PMC11384989; doi:10.3389/fendo.2024.1446863)
Supplement: Supplementary file 1 [file DataSheet1.zip › supplementary files/S2/GCST90200304/sensitivity-analysis.pdf]

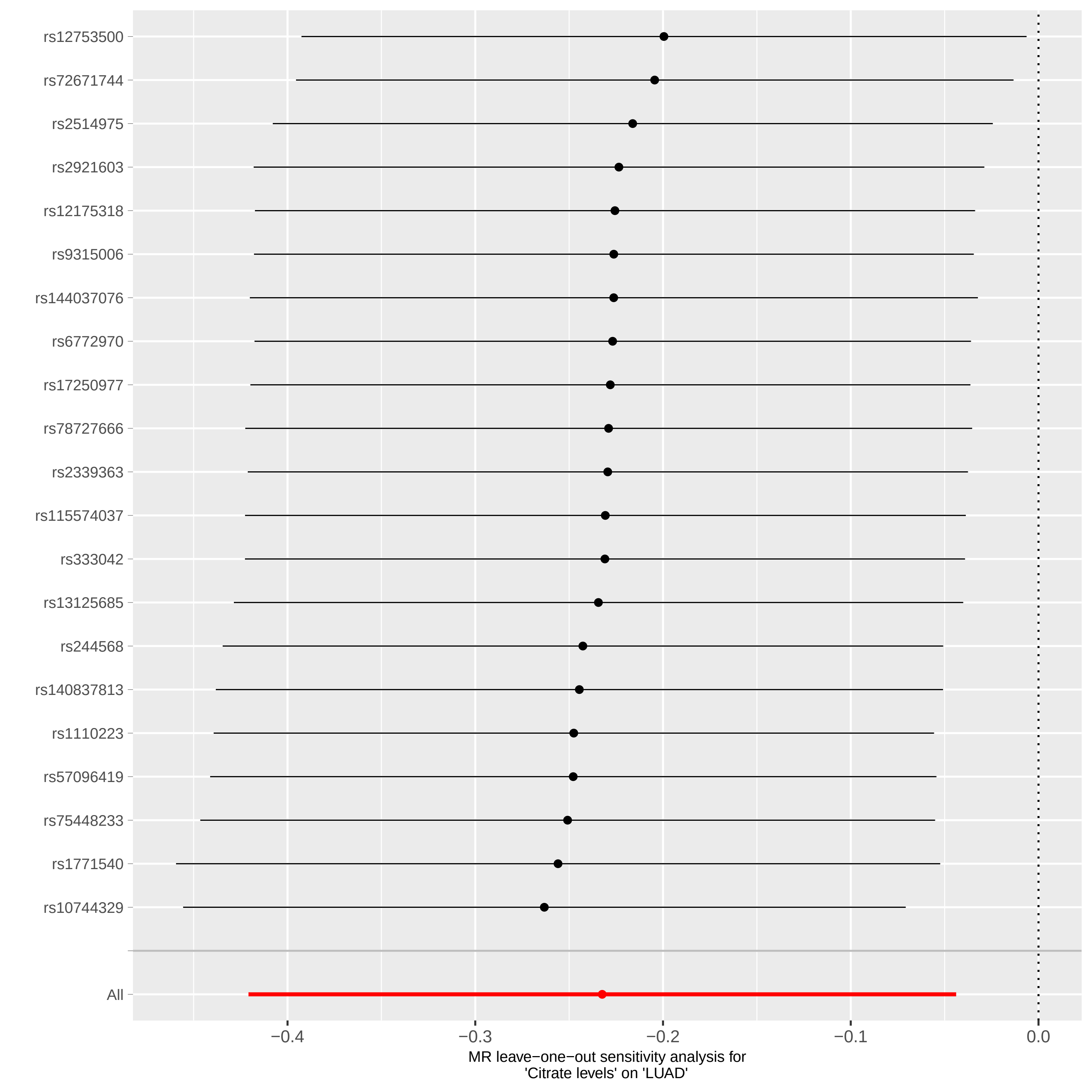

rs12753500
rs72671744
rs2514975
rs2921603
rs12175318
rs9315006
rs144037076
rs6772970
rs17250977
rs78727666
rs2339363
rs115574037
rs333042
rs13125685
rs244568
rs140837813
rs1110223
rs57096419
rs75448233
rs1771540
rs10744329
All
−0.4
−0.3
−0.2
−0.1
0.0
MR leave−one−out sensitivity analysis for
'Citrate levels' on 'LUAD'
